# Supplementary material for: The interactome of intact mitochondria by cross-linking mass spectrometry provides evidence for coexisting respiratory supercomplexes
Source: Mol Cell Proteomics. 2017 Dec 8;17(2):216–32. doi: 10.1074/mcp.RA117.000470 (PMC5795388; doi:10.1074/mcp.RA117.000470)
Supplement: Supplemental Data [file supp_RA117.000470_133922_0_supp_23978_fzffwf.zip › spectra_annotation/mito_DR_spectra_annotation/442-1-12-1-13-1.pdf]

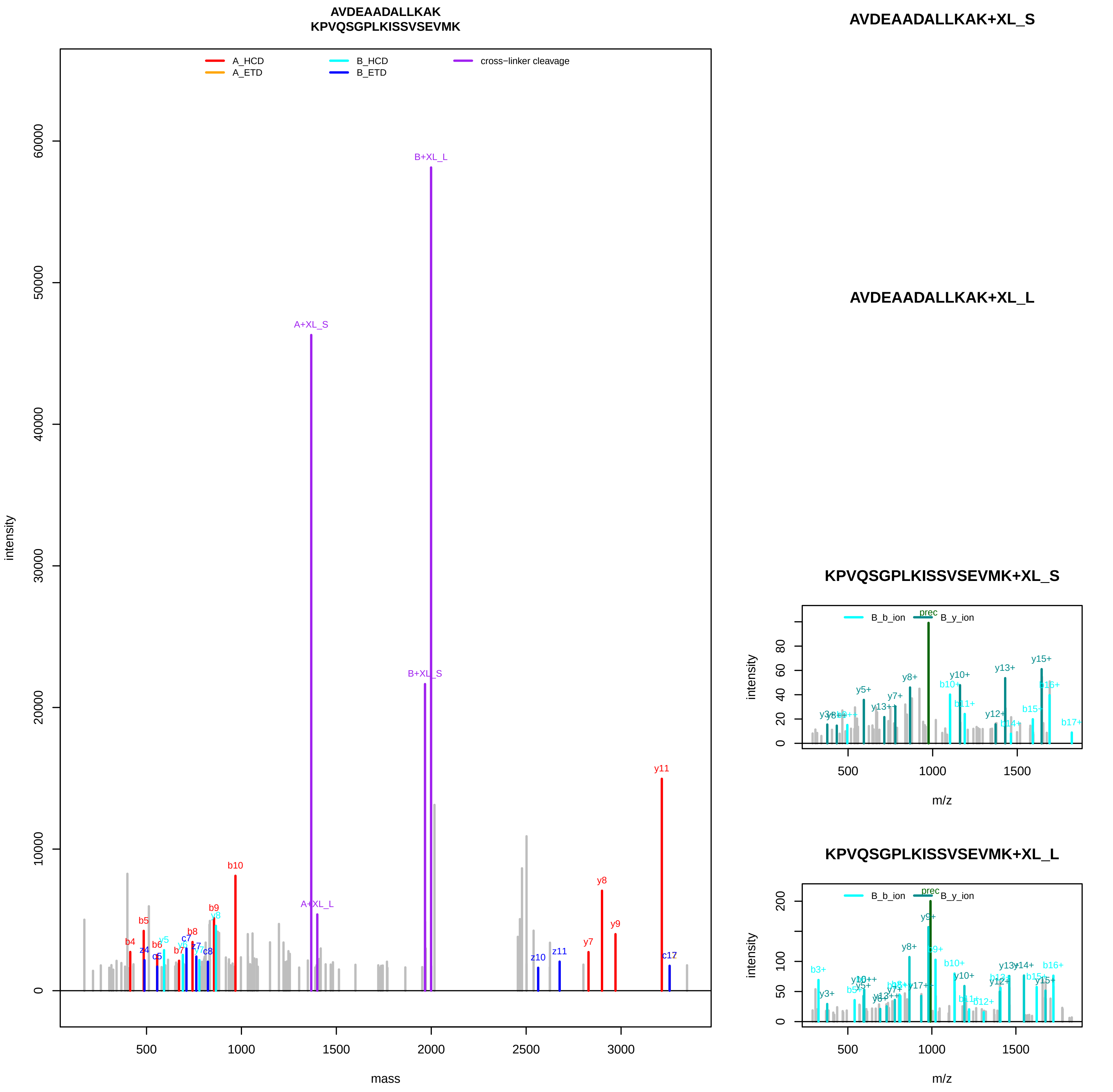
AVDEAADALLKAK
KPVQSGPLKISSVSEVMK
A_HCD
A_ETD
B_HCD
B_ETD
cross−linker cleavage
B+XL_L
A+XL_S
B+XL_S
A+XL_L
y11
y8
y9
y7
b10
b9
y8
b8
b7
b6
b5
b4
c7
z7
c8
z4
c5
y5
z10
z11
c17
intensity
mass
AVDEAADALLKAK+XL_S
AVDEAADALLKAK+XL_L
KPVQSGPLKISSVSEVMK+XL_S
B_b_ion
B_y_ion
prec
y15+
y13+
y10+
y8+
b10+
y5+
y7+
b16+
b15+
b17+
y12+
intensity
m/z
KPVQSGPLKISSVSEVMK+XL_L
y9+
b9+
y8+
b10+
y10+
b3+
y3+
b5+
y13+
y14+
b16+
y15+
b15+
b12+
intensity
m/z
